# Bites and Stings

**Published:** 1858-06

**Authors:** 


Bites and Stings.—As many of our readers are preparing to travel or go to the country for the summer, it may be useful to remind them, that an ounce vial of spirits of hartshorn should be considered one of the indispensables, as in case of being bitten or stung by any poisonous animal or insect, the immediate and free application of this alkali as a wash to the part bitten, gives instant, perfect, and permanent relief, the bite of a mad dog (we believe) not excepted; so will strong ashes-water.

